# Enabling local political committees to support the implementation of evidence-based practice — a feasibility study

**DOI:** 10.1186/s40814-022-01154-5

**Published:** 2022-08-26

**Authors:** Annika Bäck, Henna Hasson, Anna Bergström

**Affiliations:** 1 Department of Learning, Informatics, Management and Ethics, Medical Management Centre (MMC), Karolinska Institutet, 171 77 Stockholm, SE Sweden; 2 Center for Epidemiology and Community Medicine, Stockholm Region, 171 29 Stockholm, SE Sweden

**Keywords:** Co-creation, Feasibility, Politicians, Evidence-based practice, Implementation science, Social services

## Abstract

**Background:**

Local politicians can serve as enablers or barriers for health and social organizations to implement evidence, impacting the context of health and social service organizations. Increasing local politicians’ knowledge about, and support for, evidence-based practice (EBP) could be a way to strengthen the conditions in social service organizations for EBP. The aim of the study was to describe the development and assess the perceived feasibility, acceptability, and appropriateness of an intervention to enable local political committees to support the implementation of EBP. Furthermore, the achievement of the learning outcomes was examined.

**Methods:**

Workshops and interviews were used to co-create the intervention with social service representatives (*n* = 8) and local politicians (*n* = 6). A single-arm, non-blinded feasibility study was conducted in a social welfare committee with local politicians (*n* = 14) and representatives from social services (*n* = 4). Interviews and pre-post questionnaires were used to assess the intervention’s feasibility, acceptability, appropriateness, and learning outcomes. Progression criteria was set to > 80% of respondents judging the intervention to be feasible, acceptable, and appropriate. Thematic analysis and descriptive statistics were used for analysis.

**Results:**

The quantitative and qualitative results indicate that the intervention was perceived as acceptable and appropriate. However, the progression criteria for feasibility were not fully met. Qualitative findings show that the intervention was perceived as interesting, fun, and created curiosity to learn more about EBP. The discussions between the committee and the representatives from the social services department were much valued.

**Conclusions:**

Careful anchoring of the intervention and comprehensive local adaptation regarding delivery format will be central to the delivery of this intervention if offered elsewhere. Furthermore, we recommend that skills training during the intervention should be included. The collaboration between local politicians and representatives from the social services department was a vital aspect of the intervention and should not be excluded. Collaboration between these actors will be of significance in further developing support for EBP implementation, as expressed by the interview participants.

**Supplementary Information:**

The online version contains supplementary material available at 10.1186/s40814-022-01154-5.

## Key messages regarding feasibility

- What uncertainties existed regarding the feasibility?
  During the co-creation process, stakeholders highlighted some uncertainties concerning the intervention: time available for local politicians to participate in the intervention, great potential variation regarding knowledge about EBP among the politicians, and possible variation in their interest for EBP.
- What are the key feasibility findings?
  The intervention was well received and created curiosity about EBP, and the progression criteria for intervention acceptability and appropriateness were met. The progression criteria for feasibility were not fully met, indicating a need for some intervention adaptations to promote participation.
- What are the implications of the feasibility findings for the design of the main study?
  Our findings show that the political committee’s leadership, as well as the social services department, need to be invested and interested in the intervention for successful delivery. Therefore, anchoring the intervention prior to delivery in the committee and department will be crucial if the intervention is to be offered elsewhere, as will continuous adaptions in the delivery format according to committee needs. Furthermore, adding skills training to the intervention is recommended.

## Background

Research utilisation and evidence-based practice (EBP) are critical for health and social services [1]. Research utilisation has broadly been defined as the use of research findings in any aspect of one’s work [2], whilst EBP integrates research evidence, professional expertise, and client preferences and circumstances in decision-making [3]. Policymakers in health and social services are involved in research utilisation mainly in two different ways. Firstly, policymakers can be users of research evidence [4–6]. Secondly, policymakers can serve as enablers or barriers for health and social organisations to implement EBP, impacting these organisations’ inner and outer contexts [7].

Research on the first aspect—policymakers own research use in policymaking—focused on how research evidence is accessed and used and the way interventions aim to increase policymakers’ capacity to use research [4, 6, 8, 9]. Research evidence in policymaking is one of many sources for decision-making [8–10], and the use of evidence has been suggested to belong to three archetypes: instrumental, conceptual, and symbolic [2]. Instrumental use is the direct application of research findings to solve a problem, conceptual use is a more diffuse and indirect use of research to inform decision-making, and symbolic use is described as the use of research as a political weapon [11]. Although policymakers are likely to apply all three types of research use, conceptual use has been the most common in government agencies and rated as the most important [11]. Sandberg, Persson, and Garpenby (2019) found that policymakers used evidence from national clinical guidelines foremost in a legitimising fashion in negotiations and to a lesser extent in an instrumental way, for example resource allocation [12].

The second aspect of how policymakers are involved in research utilisation is their role in shaping the context for health and social services. This aspect is less researched but is based on the premise that professionals are affected by the organisational and systemic contexts, and policymakers may provide motivation, authority, credibility, and ongoing support for implementing EBP [13]. Thus, policymakers, such as local politicians, may affect the possibilities of health and social service organisations to implement EBP. For instance, political interest in research use was correlated to higher levels of research use in health organisations [14], and social service managers wanted more encouragement, support, and follow-up from the political leadership regarding the EBP implementation [15, 16]. Furthermore, managers in health care expressed that they could not be fully compliant to evidence-based guidelines without political investment [17].

As in many other countries, Sweden is governed by a multi-tier organisation at national, regional, and local levels [18]. Authorities at the regional and local levels have strong autonomy protected by the constitution [19]. Thus, they have a potentially strong role in implementation, but some studies indicate that policymakers at regional and local levels are quite passive in supporting EBP, including the implementation of evidence-based guidelines [15, 17, 20, 21]. The lack of involvement in research utilisation and EBP could be due to a lack of knowledge or interest. For instance, politicians in local government in a previous study were relatively unaware of EBP, disagreed about what role they should have in implementing EBP, and voiced a need for more guidance and support if they were to enable EBP implementation [15]. Others have found a lack of awareness among politicians about evidence-based guidelines in social services [22]. A report from the National Board of Health and Welfare stated that the interest among politicians in local government for EBP is low, according to social service managers, and could be on the decline [23].

Another barrier to research utilisation is lack of, and access to, relevant research [5, 6]. Furthermore, research evidence is not always easily applied in policymaking because many studies do not give enough information about the context for policymakers to know if the research results are applicable in their setting [8]. The same problem has been found in studies of local government, where several studies have shown that local data might trump research evidence [8, 10, 24]. Armstrong et al. (2014) found that locally derived data, such as community views, local population data, and policies, were more influential on policymaking in local government than findings from research [10]. Similarly, Atkins et al. (2017) found that local data were seen as more applicable than national evidence-based guidelines, and that local evidence even replaced evidence-based recommendations when these two conflicted. According to Atkins et al., understanding of the research evidence’s relevance needs to be developed in local government, and evidence-based guidelines need to be adapted to local contexts [24]. Local data, such as performance data or patient narratives, can contribute to EBP alongside research evidence, professional expertise, and experiences from clients/patients. Collecting and using local data need not be a threat to using research evidence, but using the different types of evidence needs to be transparent and made explicit [25].

In sum, there are barriers to local politicians’ use of research and support for EBP, which risks diminishing the contextual support for implementing EBP in social service organisations. Increasing local politicians’ knowledge about, and support for, EBP in social services could be a way to strengthen the conditions for EBP in social service organisations.

## Methods

### Aim

The study aimed to describe the development and assess an intervention’s perceived feasibility, acceptability, and appropriateness to enable local political committees to support EBP implementation. Furthermore, the learning outcome achievement was examined.

### Intervention development

There has been a move from seeing research utilisation as a linear process, in which policymakers, professionals, and service users are seen only as knowledge recipients, to a dynamic process in which knowledge is created in collaboration with stakeholders [26, 27]. Regarding interventions, benefits of co-creation include tailoring an intervention to the participants’ reality, compared to theoretically designing what ‘should’ work and to increase the chance of intervention sustainment [28]. Co-creating interventions is another way to try to ensure intervention ownership among stakeholders and to create a good fit between intervention and organisation [29]. In the current study, we adopted a structured co-creative approach [29] combined with a theory-based approach [30] to develop an intervention to enable local political committees to support EBP implementation in social services with stakeholders.

In the co-creative approach, we followed a structured process outlined by von Thiele Schwarz et al. (2018) to determine the intervention’s learning outcomes and content [29]. We started with co-creating the learning outcomes with politicians in local government and social services managers and professionals in two workshops (first with social service professionals [*n* = 5] and then with local politicians [*n* = 3]). Furthermore, face-to-face interviews were performed with social services managers (*n* = 3) and local politicians (*n* = 3); one of the interviews was performed simultaneously with a manager and a politician. During the workshop and interviews, the stakeholders were asked to reflect on two questions: What should local politicians know to lead towards EBP? What should local politicians do to lead towards EBP? Following the structured process, the workshop participants were asked to write down as many suggestions as possible on post-its. They were then asked to sort their individual suggestions into categories, followed by agreeing on appropriate headings for each category. In this way, the categories outlined a direction for the planned intervention. We needed to change the planned process for the second workshop with politicians because the participating politicians did not know what EBP entailed and found it hard to answer the two questions. Instead, notes were taken as the politicians discussed their roles in relation to the social services department. The authors used findings from the workshops and interviews to determine the intervention’s learning outcomes. Some themes from the workshops and interviews were not included in the learning outcomes because they were too large for a short intervention. Table 1 exemplifies how the interview and workshop results were summarised to form learning outcomes.

**Table 1:** Example of how co-creation workshop and interview results were summarised to form learning outcomes

Theme from workshop	Related themes from interviews	Learning outcomes
Knowledge E.g. know the basics of EBP and the factors that enable and support EBP	Understanding what EBP is E.g. the three parts of EBP, prerequisites for working with EBP, and why to use EBP	Understand what EBP entails and what affects its implementation
Who does what? Clarify roles E.g. letting evidence be part of political steering, be able to pose questions to social service officials, having trust in the profession	Knowing the roles and responsibilities of committee members versus social service officials E.g. request follow-up, ask relevant questions that are not to detailed, work with user influence	Describe the role of the committee in implementing EBP Understand why follow-up is important and be able to ask relevant questions about the results of social services

Thereafter, the intervention content was developed. The content was based on the learning outcomes outlined above and our previous findings concerning barriers for local politicians to support EBP implementation in social services (see Table 2, column 1; [15, 16, 21]). The theory-based approach [30] consisted of using implementation theories to map the barriers to supporting EBP into possible content and the choice of appropriate pedagogic methods. The barriers were first mapped according to the COM-B model, which states that behaviour is dependent on three main dimensions: capability, motivation, and opportunity [31]. Thereafter, the barriers were refined in the domains in the Theoretical Domains Framework [32]. This framework is derived from behaviour change theory with the aim to understand implementation and behaviour problems [32]. We recognised that the domains of *knowledge and skills*, *beliefs about consequences*, and *social influences* were important aspects that we would need to target with the intervention (see Table 2, column 3). In the last step, the Behaviour Change Wheel [31] was used. The Behaviour Change Wheel provides a system for designing and evaluating behaviour change interventions and proposes behaviour change techniques based on identified barriers to behaviour change [33]. We took inspiration from the possible behaviour change techniques (see Table 2, column 4) to create the intervention activities.

**Table 2:** Barriers for politicians’ support for EBP identified in interviews mapped onto the TDF and BCW

Barriers	COM-B dimension	Theoretical domains framework	Possible techniques from the behaviour change wheel
Politicians might be unaware of EBP and the actions they can take	Capability	Knowledge and skills	Information about antecedents (for EBP) Instructions on how to perform the behaviour
Politicians might be unaware of the consequences of their actions	Motivation	Beliefs about consequences	Information about social and environmental consequences
Politicians might not regard EBP as part of their mission or not know if their actions are desired. They might need social support when taking action	Opportunity	Social influences	Social comparison Social support

We also asked for feedback concerning the intervention setup from representatives of the Swedish National Board of Health and Welfare, the Swedish Agency for Health Technology Assessment and Assessment of Social Services, the County Administrative Board in Stockholm, and research and development units in Stockholm. The intervention gained interest and was well received. We received valuable information concerning the practical aspects of delivering the intervention. Another finding from the development process was that there was agreement among stakeholders in the co-creation approach that it was important to include representatives from the social services with the politicians in the intervention, as the collaboration between the two is essential. Therefore, we decided that representatives from social services would be included in the intervention, but they would not be the intervention’s primary target group.

### Design

Mixed methods, pre-post questionnaires, and interviews were used in this single-arm, non-blinded feasibility study.

### Setting

The study took place in a local government committee responsible for social services in a municipality in Stockholm County, Sweden. The municipality is classified as a commuter municipality close to a metropolitan area, as more than 40% of the working population commutes to work in the metropolis [34]. In Sweden, most social services are the municipalities’ responsibility [35]. The municipalities have significant discretion in the organisation, aim, and scope of services provided. Most politicians in local government are laypersons, doing their political assignments part-time [36]. The chair of one social welfare committee was interviewed in the intervention’s development phase of the intervention and showed interest in participating in the intervention. Therefore, local politicians in the social welfare committee were invited to participate in the feasibility study with representatives from the social services department, for which the committee is responsible. Before the intervention started, a meeting was held in March 2020 with the social welfare committee chair and the social services department’s head to discuss the proposed learning outcomes in relation to the participants’ needs to co-create the intervention. A suggestion from the workshop leaders to meet with additional politicians in the committee before the intervention to discuss intervention content was declined by the social welfare committee chair and the social services department’s head. In April 2020, the workshop leaders visited a committee meeting to briefly inform about the intervention that was planned. The workshops were executed in September and November 2020, in conjunction to, and at the location for, the committee’s regular meetings. Two of the authors (ABe, ABä) held the initial meeting, visited the committee, and were workshop leaders in the intervention. These two authors are working in a research and development unit, providing support on implementation and evaluation to health and social services, and are trained in implementation practice. They are also researchers in implementation science and have educational backgrounds in public health and medicine.

### Intervention

**Fig. 1:**
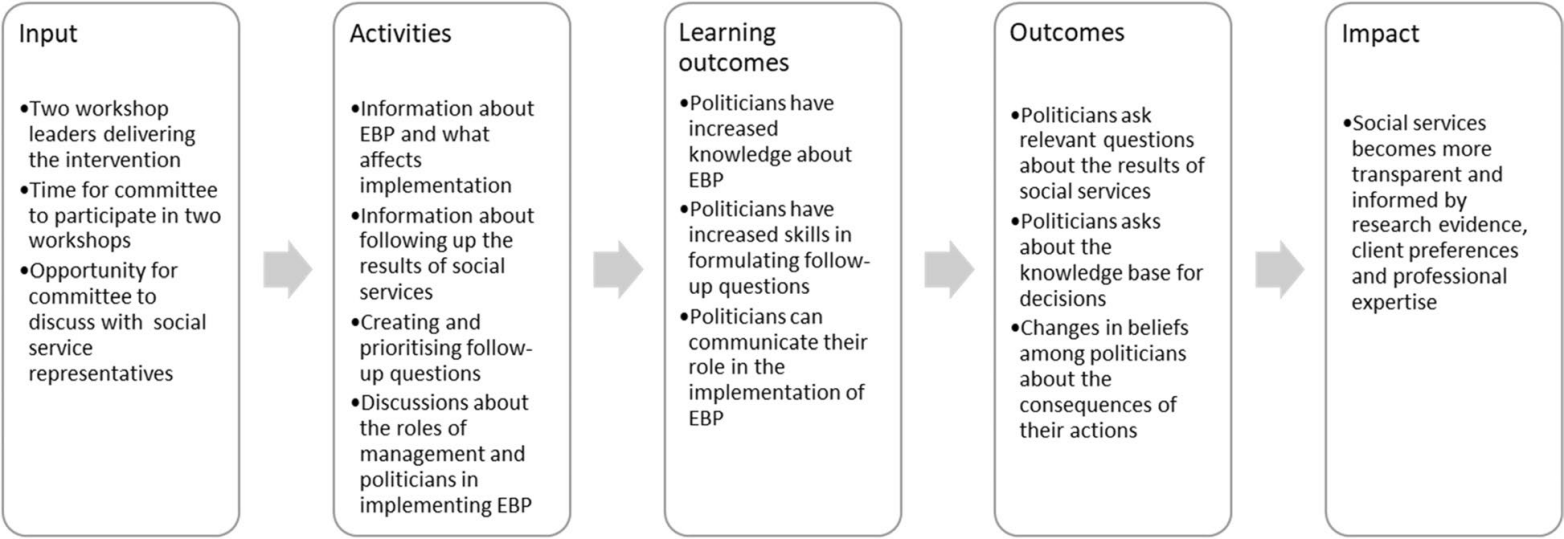
Logic model of the intervention

The intervention consisted of two workshops targeting a local political committee responsible for social services. Provided the emphasis on including social service representatives as highlighted by many in the co-creative development of the intervention, representatives from the social services department were included in the intervention to enable discussions between the political committee and the social services department as intervention activities to encourage a collaboration regarding EBP. The intervention’s logic model is outlined below (see Fig. 1). The TIDieR (template for intervention description and replication) checklist [37] was used in the intervention description (see Additional file 1).

*Workshop 1* was performed face to face in connection to a committee meeting and lasted 2 h. The workshop consisted of three parts:

1. Short lecture about EBP, what affects the implementation of EBP, and what other local government committees report doing to support EBP implementation
2. Discussion on the participants’ role as a political committee in supporting the implementation of EBP in social services
3. Discussion on the importance of continuous follow-up by the political committee of the work undertaken by the social services department and formulating relevant questions to pose

The presentation and a summary of the discussions and formulated questions were then sent to all participants. The lecture material (in Swedish) is available on reasonable request. After the first workshop, the two workshop leaders met the social welfare committee chair and the social services department’s head to discuss the content, timing, and length for the second workshop.

*Workshop 2* was held in connection to a committee meeting and undertaken digitally because of Covid-19-related restrictions. Because of the discussions with the social welfare committee chair and the social services department head, the 1-h workshop entailed three parts:

1. Summary of the work done in the first workshop and a reflection regarding what had happened between the two workshops
2. Presentation from the social services department about their current work concerning EBP
3. Discussion on how to keep supporting EBP implementation

### Characteristics of the intervention participants

**Fig. 2:**
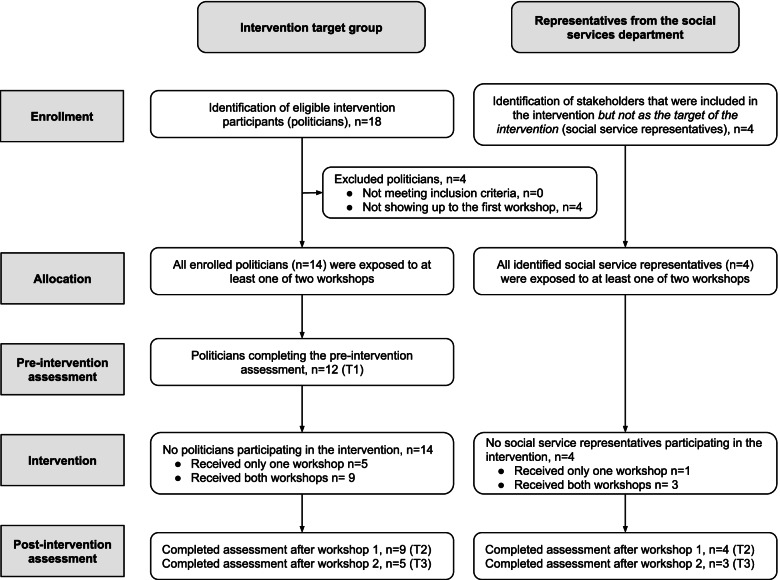
Flow diagram of participants in the intervention

The intervention target group consisted of a political committee with 18 committee members, including substitutes, eight women, and 10 men. The representatives from the social services department were three women and one man (see Fig. 2).

The politicians that answered the pre-intervention survey (*n* = 12) had been active in the present committee for an average of approximately 2 years, and 50% had a university degree. The four social services department representatives included the department head, a middle-level manager, and two social service professionals working with quality assurance and user influence.

### Measures

#### Progression criteria

Progression criteria may be used to evaluate whether to proceed from a feasibility study to an evaluation study [38]. Table 3 presents the progression criteria we formulated before the intervention started.

**Table 3:** Progression criteria

Progression criteria	Measures used	Assessment of criteria
Feasibility of recruitment	Percentage of eligible politicians participating in the intervention and percentage of enrolled politicians participating in both workshops	> 60% of the politicians participate in the intervention, and > 60% of enrolled politicians attend both workshops of the intervention
Feasibility, acceptability, and appropriateness of intervention (quantitative)	Percentage of politicians and representatives from the social services department quantitatively reporting the intervention to be feasible, acceptable, and appropriate	> 80% of politicians and representatives from the social services department judge the intervention feasible, appropriate, and acceptable with ratings ≥ 51 (on a scale of 0–100)
Feasibility, acceptability, and appropriateness of intervention (qualitative)	Qualitative results from interviews with politicians and representatives from the social services department regarding feasibility, acceptability, and appropriateness of intervention components, content, and delivery	Judgement by the research group together with the other measures

#### Feasibility, acceptability, and appropriateness

Feasibility, acceptability, and appropriateness were measured by a selection of six adapted items (see Table 4) from the *Acceptability of Intervention Measure*, *Intervention Appropriateness Measure,* and *Feasibility of Intervention Measure* [39]. The items were adapted to fit the current intervention better, which is not an evidence-based method used by professionals, but an intervention targeting a political committee. The response scale was a visual analogue scale from 0–100 (0 = strongly disagree, and 100 = strongly agree).

Feasibility, acceptability, and appropriateness were also explored in interviews with politicians and representatives from the social services department. The semi-structured interview guide explored issues related to intervention content and delivery, such as feasibility and acceptability of intervention components, need for development, perceived value, and unintended consequences [40].

#### Learning outcomes

Learning outcomes for the politicians were the intermediate outcomes of the intervention as presented in Table 5. The response scale was a visual analogue scale from 0–100 (0 = to a very small extent, and 100 = to a very large extent). Learning outcomes were also explored in the interviews.

### Data collection

Data concerning feasibility, acceptability, and appropriateness were collected from the intervention group (politicians) and the social services department representatives. The data on learning outcomes was only collected from the intervention group (politicians). Quantitative data were collected through web surveys pre-intervention, T1 (learning outcomes); post-intervention after workshop one, T2 (feasibility, acceptability, appropriateness, and learning outcomes); and after workshop two, T3 (feasibility, acceptability, appropriateness, and learning outcomes). Reminders at T1, T2, and T3 were sent out one, two, and four times, respectively.

All participants, politicians and representatives from the social services department, who had participated in at least Workshop 1 were invited to a semi-structured interview. Three politicians and two representatives from the social services department agreed to be interviewed. Interviews were performed digitally November 2020–January 2021. The interviewer was not involved in the development or execution of the intervention.

### Analysis

The quantitative data were analysed in SPSS 25 using descriptive statistics. The qualitative data was analysed using inductive thematic analysis [41]. The interviews were transcribed verbatim, and each sentence or text passage relevant for the research aim was first sorted based on the research aim’s dimensions: feasibility, acceptability, appropriateness, and learning outcomes. The first and last author simultaneously sorted one of the interviews, discussing and resolving any discrepancies regarding the sorting. Next, the first author generated initial inductive codes for all sentences or text passages. This coding was discussed between the first and last author; some minor revisions were made; and some codes were dropped from the analysis because they did not relate to the research aim. After finalising the coding, the codes were sorted into tentative themes. After assigning all codes to a theme, the themes were reviewed to ensure that data in each theme were not too diverse, and that themes were distinctly separate from others. The reviewing process was also discussed between the first and last author. Finally, each theme was named and described. All authors discussed the names and descriptions of the themes. Each theme highlights some relevant aspect of the research aim but does not necessarily contain all interview participants’ codes. In addition to the themes identified in the analysis, some contextual information was not related to the intervention itself but could be of importance in the interpretation of the results. This contextual information was coded separately and presented alongside the results. All the interview participants have been quoted in the results; however to protect the anonymity of the participants, we have chosen not to indicate what quotes belong to which participant.

## Results

The progression criteria for feasibility for recruitment were met because the percentage of eligible politicians participating in the intervention was 78%. Furthermore, 64% of those participating in the first workshop also attended the second. Figure 2 presents the number of participants in the intervention.

Regarding the progression criteria for acceptability and appropriateness, more than 80% of respondents reported the intervention to be acceptable and appropriate at T2 and T3, whereas less than 80% reported the intervention to be feasible at both time points. Thus, the progression criteria for quantitative reporting for intervention feasibility were not fully met.

### Quantitative results: feasibility, acceptability, appropriateness, and learning outcomes

The quantitative results showed that the participants, both politicians and social services department representatives, were satisfied with the intervention. For both time points, the respondents mean ratings of feasibility, acceptability, and appropriateness were greater than 70 (see Table 4).

**Table 4:** Politicians’ and representatives’ ratings of the primary outcomes: acceptability, appropriateness, and feasibility (scale: 1–100, *M* mean values)

**Dimension**	**Primary outcomes** *Politicians and representatives*		**T2** *n* = 13	**T3** *n* = 8
Acceptability	The training meets my approval	M (SD)	86 (9)	81 (19)
Acceptability	I like the training	M (SD)	88 (9)	82 (17)
Appropriateness	The training seems suitable for the social welfare committee’s work	M (SD)	87 (15)	83 (18)
Appropriateness	The training seems applicable for the social welfare committee’s work	M (SD)	83 (17)	80 (19)
Feasibility	The training seems implementable in the social welfare committee	M (SD)	84 (17)	78 (25)
Feasibility	The content of the training seems easy to use	M (SD)	72 (21)	76 (18)

The quantitative assessments of the learning outcomes were collected from only the politicians. The quantitative results showed that the mean values were higher on all but one item measuring the intervention’s learning outcomes from T1 to T3. The exception was the item concerning requesting follow-up of social services results (see Table 5).

**Table 5:** Politicians’ ratings of the intervention’s learning outcomes (scale: 1–100, *M* mean values)

**Learning outcomes** *Politicians only*		**T1**	**T2**	**T3**
I have knowledge about the three parts of EBP	M (SD)	39 (25)	79 (19)	91 (17)
I have knowledge about how to support the implementation of EBP	M (SD)	39 (24)	66 (21)	73 (15)
My role as a committee member in the implementation of EBP is clear	M (SD)	42 (23)	61 (23)	71 (20)
I have knowledge about what systematic follow-up entails in social services	M (SD)	44 (34)	70 (27)	68 (21)
I request follow-up of the social services results	M (SD)	75 (24)	67 (33)	74 (19)
I ask questions to the department’s representatives about the social services results	M (SD)	49 (27)	63 (30)	74 (17)

Note: The number of respondents at T1 varied between 8 and 12 for the different items, whereas 9 and 5 individuals responded to the survey at T2 and T3, respectively

### Qualitative results: feasibility, acceptability, appropriateness, and learning outcomes

**Fig. 3:**
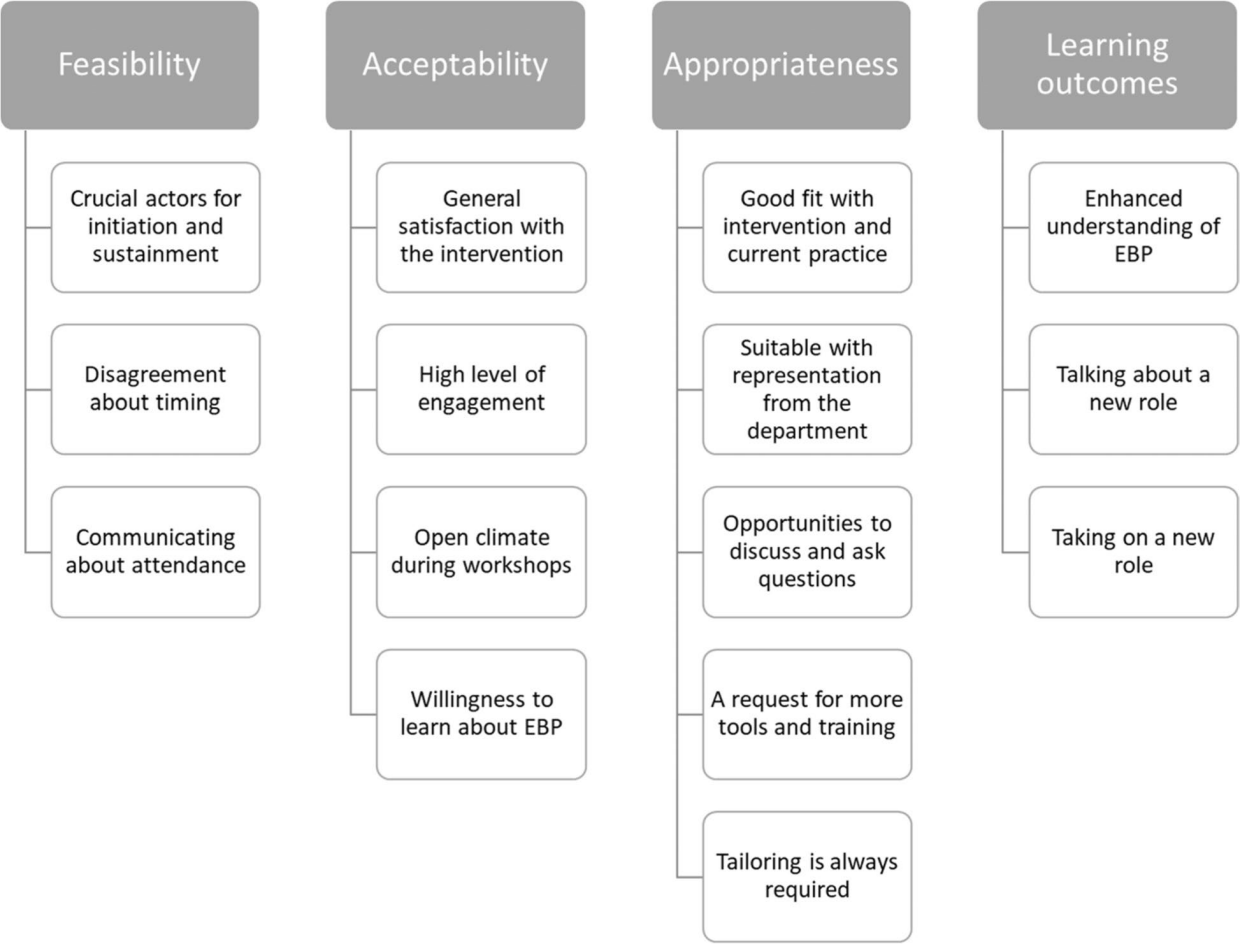
Overview of themes identified in the interviews

The qualitative analysis generated 15 themes related to the intervention’s feasibility, appropriateness, acceptability, and learning outcomes (see Fig. 3).

#### Feasibility

In the theme *crucial actors for initiation and sustainment*, the interview participants pointed out several key actors of the intervention without whom the intervention would have been difficult to execute, and the same actors were pointed to as essential for the integration of new ways of working after the intervention. The actors were the committee chair, the praesidium, the social services department head, and the personal social services manager. One interview participant described crucial actors as gatekeepers for recruitment: ‘After all, this is all so dependent on who you talk to. If it is a committee chair, or praesidium, or head of a department who thinks that “No, no, no, we do not have time for that”, then it probably doesn’t matter how interesting your approach is’.

The theme *disagreement about timing* concerned the conflicting views expressed about the best time for the workshops and their durations. Most participants wanted the intervention to take place in connection to official committee meetings in some form, whereas a few wanted to have the workshops on separate days. Some interview participants felt that the intervention’s extent was sufficient, whereas others wanted longer workshops or for the workshops to have a less-condensed schedule. ‘Well, it’s possible you should have had a third time, in order to somehow be able to do an evaluation together in peace and quiet. How do we move forward now? What have you thought of these two times you had? Maybe they [workshop leaders] had it on the last one, but I remember the last time, the workshop leaders were a little stressed’.

Regarding *communicating about attendance* in the workshops, it was highlighted that information about the upcoming intervention needed to be provided to the committee and the department with great foresight to ease planning for the participants. It was also viewed positively if the information came from the head of social services and the committee chair.

#### Acceptability

The interview participants expressed a *general satisfaction with the intervention*, being overall content with its main components, format, and execution. The face-to-face format was preferred to the digital format: ‘I think there are advantages of being as many as possible on site. This distance thing in all glory, I myself work like that fairly much, but it won’t be quite the same thing as being physically present’. One participant voiced that the intervention had not been ‘anchored’ at the department before the intervention’s start, creating hesitation from the participant at first, but that this hesitation had disappeared during the intervention: ‘I have only positive things to say. It turned out better than I thought – because I was incredibly suspicious from the beginning, I can tell you’.

There was an *open climate during workshops* consisting of an open-minded environment where participants could pose questions without feeling constrained as well as a positive atmosphere. Interview participants described a *high level of engagement* in that the workshop leaders were engaging and encouraging by listening and asking questions, and that the topic created interest and curiosity. Furthermore, it was described that those attending the workshops were actively engaged in the discussions, and that exchanging views was interesting and fun, although easier in the physical format than the digital.

The theme *willingness to learn about EBP* depicted interview participants’ descriptions about learning more about EBP. Some interview participants highlighted a need for a deepened knowledge about EBP for the praesidium. Others stated that they wanted recurring training in EBP for the entire committee and follow-ups of what had been learnt to avoid forgetting: ‘But I think this is the kind of thing you need to be reminded of. We’re going to have a follow-up, and I think that’s very good. And then I think it should really be a recurring education […] maybe at the beginning of a term of office’. However, interviewed politicians also stated that they had not engaged with the material produced in the workshops after the end of the intervention.

#### Appropriateness

In the theme *good fit with intervention and current practice*, interview participants described that the intervention was perceived as relevant and fit their needs. Posing questions to the department was a natural way of working for the committee. The department welcomed relevant questions from the committee and wanted to utilise the intervention’s content: ‘It’s great because it also strengthens us in continuing with the development issues we have and being more stringent when explaining things because politics can answer back then “Well, how – what have you been thinking about this? How do you combine competency with the evidence-based interventions? Why do you say that?” Yes, it creates another—a more professional—conversation between us and politics’. The politicians described that they intended to discuss how the use of relevant questions might be best organised in future committee work. At the same time, the politicians stated that they believed that social services already were working according to EBP to a great extent.

The interview participants expressed appreciation for the *opportunities to discuss and ask questions* about EBP during the workshops rather than only obtaining information about EBP. The opportunity to collectively discuss how to proceed with supporting EBP was described as useful, and several interview participants mentioned that the documented discussions had provided tools for further EBP development: ‘It is also a very good piece of the discussion because then, nowadays, there are possibilities to looking back on it and, yes... see what we agreed on’. Being able to pose questions to the workshop leaders was appreciated because the committee’s knowledge about EBP was fairly limited.

Although interview participants expressed that the discussions had provided them with tools for EBP development, the politicians also voiced a *request for more tools and training* on asking relevant questions, how to act as a committee in relation to EBP, and how to review cases presented to them by the administration. One idea was to include case-reviewing skills training in the intervention.

The theme *suitable to have representation from the department* concerned interview participants’ perceptions that involving representatives from the department in the intervention was pertinent. This way, everyone got the same information, and several different experiences and perspectives were highlighted. These types of discussions between the committee and department were rare and allowed the politicians to pose direct questions to the department, which had local expert knowledge.

*Tailoring is always needed* contained interview participants’ suggestions on how to best tailor the intervention to the particular committee’s needs before the intervention’s start. It was stated that it was good to undertake a formative evaluation to enable adaptation of, for example, the intervention’s difficulty level. Furthermore, it was highlighted that more information could be given about EBP’s relevance and benefits prior to the intervention to motivate the politicians to participate in the intervention, and that it is important to engage the chair of the committee and the head of social services from the start to ensure their mutual interest in and commitment to participation.

#### Learning outcomes

Regarding what had been learnt during the intervention, interview participants described an *enhanced understanding of EBP* among the politicians. The intervention increased knowledge about EBP, mainly on a conceptual level. Several interview participants said it became clear that EBP entailed three parts and meant a new way of thinking, and that the intervention had given them new terminology for things the department previously had been working with without calling it EBP. One politician described this parallelism as follows: ‘I just needed some time to figure out that these things were connected […] it took a little while to understand it, and there were several who had an *aha* experience when I asked the question, I think. That evidence-based practice was all these areas described; I think it was three bubbles or the like’.

The theme *talking about a new role* encompassed descriptions of the committee’s role in supporting EBP’s implementation following the intervention and whether this role had changed. Several perspectives of the committee’s role were highlighted and varied with the committee position. One politician described the role of the committee as follows: ‘Yes, so what we can do in our role is to follow up on how the work is going, that you give some assignment, we can commission. We can pose questions; we can discuss. And then, it’s kind of at the committee meetings that the committee, if we speak of the committee as a whole, might pose the right questions’. The committee chair was perceived to have a specific responsibility to sustain the intervention’s content and act as a champion for EBP. The politicians expressed having a new perspective when reading cases from the department after the intervention. However, there was still some uncertainty among the politicians regarding supporting EBP in more concrete terms as a political committee.

In the last theme, *taking on a new role*, representatives from the department stated, ‘There have been more questions; there are more like active questions in the committee,’ and that more politicians posed these questions, for example regarding the evidence base for certain methods. However, related to some politicians’ uncertainty regarding how to support EBP implementation in practice, described in the previous theme, the interviewed politicians perceived that they had not begun posing relevant questions based on intervention content, and the intervention had not changed their way of working at the time of the interview. They felt a need for more tools and training to pose these questions and a need to work together as a committee and in collaboration with the social services department in further development of EBP support.

#### Context

The interview participants described that social welfare committees generally have lower levels of political conflict than committees in other sectors. Furthermore, the collaboration between the social welfare committee and the social services department and among the political parties in the committee was described as especially highly functioning.

## Discussion

The quantitative and qualitative results indicate that the intervention to enable local political committees in supporting EBP implementation was perceived as appropriate and acceptable by the politicians and the participating social service representatives. Furthermore, the examined learning outcomes seem to have been achieved. Regarding the intervention’s feasibility, the quantitative progression criteria were not fully met. If the intervention were to be offered elsewhere or assessed in an evaluation study, three vital aspects to consider are outlined below. Thereafter, the findings are discussed in relation to prior research.

First, the crucial roles of the committee chair and the head of the social service department cannot be underestimated. Their interest in participating in the intervention is likely to determine both whether it can be carried out at all and the extent to which the learning will be utilised in decision-making. Furthermore, it can be beneficial to involve them early in the development and planning process to ensure anchoring of the initiative in the committee as well as in the department.

Second, including skills training as a pedagogic approach could further help the politicians in acquiring skills in asking relevant questions. Due to time constraints, skills training was not part of this intervention but was mentioned in the interviews as a potentially valuable component. Skills training during an intervention could include reviewing concrete cases, both clients’ cases and strategic planning, presented by the social services department. The fact that the interviewed politicians did not perceive that they had changed their behaviour in posing questions could be partly because no skills training was delivered during the intervention. Furthermore, although knowledge is a prerequisite for behaviour change, knowledge alone is however not enough for behaviour change to occur [31]. Thus, in scaling up this intervention, it is essential to develop appropriate and feasible measures to assess behaviour change.

Third, there is a need for intervention developers to be flexible because the preferred format regarding intervention scheduling and duration might vary among committees. Some participants voiced that more time during the workshops would make room for more reflection and discussion. This desire was also noticed in the second workshop, when the discussion on future development of the committees’ EBP support needed to be cut short because of time constraints. This preference could be reflected in the quantitative ratings for the intervention’s feasibility. The ratings were just below the assessment criteria, indicating that some changes might be needed to increase the feasibility. For instance, making the second workshop longer and involving the committee and social services representatives early in the planning process could positively affect the intervention’s perceived feasibility.

Although the interest in co-creation within health service research is increasing [26], there remains a research gap regarding how to co-create interventions in practice [28]. Our study contributes to the literature describing co-creative approaches to intervention design. Although the intervention itself was perceived as acceptable, appropriate, and relatively feasible, we faced challenges in recruiting stakeholders (i.e., local politicians, managers, and social service professionals) for co-creation of the intervention. This challenge in involving stakeholders to create the intervention could be due to low knowledge about or interest in EBP among local politicians, reluctance from social service managers and professionals in involving local politicians in their EBP work, or lack of time to participate. Moreover, stakeholders might be unfamiliar with being involved in the creation of a research intervention; they might expect researchers to develop it. Furthermore, the researchers were unknown to the invited politicians and managers/professionals, which might also have influenced stakeholders to be less prone to respond to the invitation. The importance of building trustful relationships between researchers and stakeholders to enable co-creative approaches has previously been pointed out [42–44]. Once we managed to build a relationship with one municipality, the intervention’s delivery was smooth; these relationships were key to this delivery. Without the anchoring of the intervention with crucial actors, it would have been challenging to achieve buy in for the intervention.

Tailoring during the intervention was also an essential aspect found in the qualitative results. An approach to co-creation that encourages local adaptation has been highlighted as one of co-creation’s success factors [26]. Our findings show that tailoring might be useful in the delivery of an intervention as well. For instance, gathering information about the politicians’ knowledge about EBP and securing support from the social services department head and the committee chair before intervention start were deemed useful for the intervention. Time points for delivering the intervention and its duration might also require tailoring to secure high participation. The facts that interview participants voiced a request for continuous education about EBP and that some wanted deeper EBP knowledge show that intervention participation might create additional needs for more EBP knowledge, of which participants were not aware before the intervention’s start. Thus, there could be a possibility to build on the intervention following the initial two workshops.

We found that politicians’ knowledge about EBP was fairly low at the intervention’s start. This finding resonates with the results of a previous study showing that many local politicians are rather unaware about EBP [15]. Given that earlier studies further indicated that some politicians do not actively support EBP or engage in evidence-based guidelines [15, 17, 20, 21], the intervention’s positive assessments are encouraging. The participants valued that local politicians and social service representatives jointly participated, and that the workshop included discussions between these two groups. Our findings suggest that communication among those in politico-administrative leadership (politician and manager) positions could be significant in implementing EBP. In the final governmental report on a new law regulating social service organisations [45], it is recommended to introduce a demand that social service organisations provide services based on science and proven experience. This formulation would further accentuate the demands for EBP in social services. Local politicians’ role in EBP’s future development remains unclear; the report does not address this issue [45]. There are ongoing efforts to build national and regional knowledge structures in social services to enable EBP. Researchers now warn against failing to ensure the active participation of both local social services organisations and leadership in these knowledge structures [46]. We agree that local organisations’ and leaders’ participation is important in the capacity building for EBP implementation. Based on our results, we argue that active collaboration between the politico-administrative leadership could support EBP implementation. As the participants expressed in the interviews, the committees’ future support for EBP in social services must be discussed and developed between the committee and representatives from the social services department. Therefore, having the political committee and representatives participate together is a vital aspect of the intervention.

Our qualitative findings indicate that the intervention seems to be related to the conceptual use of research in that the participants mainly speak of an increased understanding of the concept of EBP and what questions they might pose to the social services department. Because the intervention aimed to enable local politicians in supporting the implementation of EBP rather than to instrumentally use research themselves, this outcome is expected. Others have sought to increase the use of evidence-based decision-making, which involves both conceptual and instrumental use of research in local government [47]. However, in the Swedish context, where local politicians execute their political responsibilities mainly outside their daily jobs, that type of complex intervention was deemed infeasible and less relevant for local politicians and the social service organisations’ needs.

### Methodological considerations and limitations

Two researchers were involved in identifying themes to enrich the qualitative analysis. This is a strength because different researchers can have different presumptions and experiences, making the analysis more nuanced. Our pre-understanding influenced our analysis about implementation theory regarding the intervention’s acceptability, appropriateness, and feasibility [48], and we noticed that the themes were interconnected and affected one another. For instance, it was sometimes difficult to determine whether certain codes were related to the themes concerning appropriateness or those concerning learning outcomes; the conceptual boundaries between these concepts are indistinct.

Limitations of this study that must be considered are the small participant sample, the surveys’ low response rate, and the relatively few interview participants. In this regard, it was beneficial that mixed methods were used to triangulate the results [49]. It is possible that intervention participants who did not answer the web surveys or take part in the interviews had different views of the intervention than those of the interview respondents. The small sample means each individual has a large impact on our findings, and it highlights the importance of using quantitative and qualitative data in these types of evaluations. Another limitation is that no significance testing has been conducted regarding the quantitative findings. Finally, this study’s findings must be seen in light of the ongoing Covid-19 pandemic. This time has been hectic for social services, as for all other welfare organisations. More specifically, regarding the current intervention, the pandemic meant that face-to-face delivery needed to shift to digital format in the middle of the intervention. This could have impacted the participants’ motivation to take part in the intervention and the evaluation. The participants were less satisfied with the digital workshop than with the physical workshop.

## Conclusions

The intervention to enable politicians to support EBP implementation was perceived as acceptable, appropriate, and relatively feasible, but the quantitative assessment criteria for feasibility were not fully met. Adaptions to the intervention are recommended based on our findings. If the intervention was to be offered elsewhere, anchoring the intervention before the start and local adaptation of the delivery format would be central to this intervention’s delivery. Furthermore, we recommend that the intervention includes skills training. If policymakers are to support and propagate EBP, they need the prerequisites to do so. Whether this intervention is the best way of providing these prerequisites must be further evaluated in future studies. The collaboration between local politicians and representatives from the social services department was a vital aspect of the intervention and should not be excluded. As expressed by the interview participants, collaboration between these actors will be significant in further developing EBP implementation support.

## Supplementary Information

**Additional file 1. **The TIDieR (Template for Intervention Description and Replication) Checklist*.

## Data Availability

To protect participant anonymity, the data sets used and/or analysed during the current study are not publicly available, but parts of the data could be provided from the corresponding author upon reasonable request.
